# Left atrial strain correlates with severity of cardiac involvement in Anderson-Fabry disease

**DOI:** 10.1007/s00330-022-09183-7

**Published:** 2022-11-02

**Authors:** Moritz C. Halfmann, Sebastian Altmann, U. Joseph Schoepf, Constantin Reichardt, Julia B. Hennermann, Karl-Friedrich Kreitner, Roman Kloeckner, Felix Hahn, Christoph Dueber, Akos Varga-Szemes, Christoph Kampmann, Tilman Emrich

**Affiliations:** 1 Department for Diagnostic and Interventional Radiology, University Medical Center Mainz, Johannes Gutenberg University, Langenbeckst. 1, 55131 Mainz, Germany; 2 German Centre for Cardiovascular Research, DZHK - Partner site Rhine-Main, Langenbeckst 1, 55131 Mainz, Germany; 3 Department of Neuroradiology, University Medical Center Mainz, Johannes Gutenberg University, Langenbeckst. 1, 55131 Mainz, Germany; 4 Division of Cardiovascular Imaging, Department of Radiology and Radiological Science, Medical University of South Carolina, 25 Courtenay Dr, Charleston, SC 29425 USA; 5 Center of Pediatric and Adolescent Medicine, Department of Metabolic Diseases, Villa Metabolica, University Medical Center Mainz, Johannes Gutenberg University, Langenbeckst. 1, 55131 Mainz, Germany; 6 Center of Pediatric and Adolescent Medicine, Department of Paediatric Cardiology, University Medical Center Mainz, Johannes Gutenberg University, Langenbeckst. 1, 55131 Mainz, Germany

**Keywords:** Cardiomyopathy, Atria, Anderson-Fabry disease, Strain, Feature-tracking

## Abstract

**Objectives:**

Cardiac involvement in Anderson-Fabry disease (AFD) results in myocardial lipid depositions. An early diagnosis can maximize therapeutic benefit. Thus, this study aims to investigate the potential of cardiac MRI (CMR) based parameters of left atrial (LA) function and strain to detect early stages of AFD.

**Methods:**

Patients (*n* = 58, age 40 (29–51) years, 31 female) with genetically proven AFD had undergone CMR including left ventricular (LV) volumetry, mass index (LVMi), T1, and late gadolinium enhancement, complemented by LA and LV strain measurements and atrial emptying fractions. Patients were stratified into three disease phases and compared to age and sex-matched healthy controls (HC, *n* = 58, age 41 [26–56] years, 31 female).

**Results:**

A total of 19 early-, 20 intermediate-, and 19 advanced-phase patients were included. LV and LA reservoir strain was significantly impaired in all AFD phases, including early disease (both *p* < 0.001). In contrast, LA volumetry, T1, and LVMi showed no significant differences between the early phase and HC (*p* > 0.05). In the intermediate phase, LVMi and T1 demonstrated significant differences. In advanced phase, all parameters except active emptying fractions differed significantly from HC. ROC curve analyses of early disease phases revealed superior diagnostic confidence for the LA reservoir strain (AUC 0.88, sensitivity 89%, specificity 75%) over the LV strain (AUC 0.82).

**Conclusions:**

LA reservoir strain showed impairment in early AFD and significantly correlated with disease severity. The novel approach performed better in identifying early disease than the established approach using LVMi and T1. Further studies are needed to evaluate whether these results justify earlier initiation of therapy and help minimize cardiac complications.

**Key Points:**

*• Parameters of left atrial function and deformation showed impairments in the early stages of Anderson-Fabry disease and correlated significantly with the severity of Anderson-Fabry disease.*

*• Left atrial reservoir strain performed superior to ventricular strain in detecting early myocardial involvement in Anderson-Fabry disease and improved diagnostic accuracies of approaches already using ventricular strain.*

*• Further studies are needed to evaluate whether earlier initiation of enzyme replacement therapy based on these results can help minimize cardiac complications from Anderson-Fabry disease.*

**Supplementary Information:**

The online version contains supplementary material available at 10.1007/s00330-022-09183-7.

## Introduction

Anderson-Fabry disease (AFD) is a rare, hereditary, X-linked, lysosomal storage disease caused by mutations in the ɑ-galactosidase A gene, resulting in an enzymatic deficiency of alpha-galactosidase [1, 2]. Accumulation of sphingolipids leads to myocardial lipid depositions. With disease progression, these structural changes may induce fibrosis, ultimately leading to heart failure and arrhythmia [1, 3, 4]. Although, enzyme replacement (ERT) and oral chaperone therapies are available their maximum therapeutic benefit is only achievable if initiated prior to irreversible organ damage or dysfunction [5–9].

Classic imaging biomarkers for advanced AFD are left ventricular (LV) hypertrophy and late gadolinium enhancement (LGE). T1 mapping on the other hand can directly visualize sphingolipid deposition in the myocardium, even in earlier disease stages [10, 11]. In addition, the analysis of myocardial deformation, known as strain imaging, has recently gained more attention in the early diagnosis of AFD [6, 12–16].

Strain analysis can be performed by speckle tracking echocardiography (STE) and cardiac magnetic resonance imaging (CMR) [17–21]. While STE is highly user-dependent and may suffer from patient-specific factors such as pour acoustic windows, CMR has become the preferred method for strain assessment due to its excellent reproducibility and accuracy, especially in regard to the atria [22–26]. Analysis of atrial function using volumetric and deformation parameters may have the potential to provide insights into the early stages of impairment in AFD, as sphingolipid accumulation in the myocardium subsequently leads to myocardial stiffening and diastolic dysfunction. Both phenomena have been linked to disturbed atrial function [13, 27].

Therefore, the goal of this study was to assess atrial function and deformation using CMR-derived total (TEF), passive (PEF), and active (AEF) atrial emptying fractions and strain analysis to address the hypothesis that these parameters allow to discriminate not only between AFD patients and healthy controls (HC) but are able to evaluate disease severity.

## Materials and methods

The protocol of this retrospective single-center study was approved by the responsible institutional ethics committee.

### Study cohort

A total of 58 AFD patients (age range 8–77 years; 5 patients under the age of 18) were enrolled and stratified into three disease severity phases based on CMR findings [4] (Table S1), as follows: phase I included patients with known disease-causing mutation but without signs of disease; phase II comprised patients with an increased myocardial mass and/or pathologically shortened T1; and phase III involved patients with shortened T1 and highly abnormal myocardial mass along with pathological LGE. Evaluation and patient stratification were performed by a radiologist with > 10 years of experience in CMR and sex-specific cut-offs were based on the healthy controls’ mean values ± 2 standard deviations (SD) for phase II and mean values ± 3 SD for phase III findings. In case of the presence of pathological LGE with a typical pattern, the patient was moved to phase III by default. In case of conflicting results from T1 and LVMi measurements, cases were advanced to the higher of the phases in question.

A group of 58 healthy subjects who were matched 1:1 with AFD patients on a basis of age and sex served as a control group. Based on their clinical history, none of the subjects have had any cardiovascular events or symptoms or cardiovascular risk factors (e.g., hypertension or diabetes). All healthy controls (HC) had normal LV volumes, normal myocardial T1 and T2 relaxation times according to institutional reference ranges, and no LGE.

### Image acquisition

All patients underwent CMR on 3-T systems (MAGNETOM Prisma or Skyra, Siemens Healthcare). Standard cardiac views (2-, 3-, and 4-chamber) were acquired using a unified imaging protocol comprising a conventional balanced steady-state free-precession cine sequence with retrospective ECG gating and standard acceleration (GeneRalized Autocalibrating Partial Parallel Acquisition, GRAPPA 3). Typical pulse sequence parameters were as follows: repetition time (TR) 3.77 ms; echo time (TE) 1.39 ms; reconstructed cardiac phases 25; field of view (FOV) 360 mm; flip angle 60°; voxel-size 1.5 × 1.5 × 8.0 mm; and cardiac cycle/slice 10.

Native T1 mapping was performed in three short-axis slices predefined from the previously acquired cine stack (basal, midventricular, and apical). A commercially available modified Look-Locker inversion-recovery sequence with a 5(3)3 scheme was applied with the following pulse sequence parameters: TR/TE 331.68 / 1.12 ms; FOV 630 × 360 mm; matrix 256 × 256; slice thickness 8 mm; flip angle 35°; bandwidth 1085 Hz/Pixel; and GRAPPA 3.

For contrast enhancement, 0.2 mmol/kg gadoteric acid (Dotarem, Guerbet) was administered. LGE images were acquired 10 min after contrast injection using a segmented T1-weighted inversion recovery ultrafast spoiled gradient echo sequence in long- and short-axis views identical to those used for cine imaging. The following pulse sequence parameters were applied: TR 2 × RR-interval, TE 4.38 ms, in-plane resolution 1.4 × 1.8 mm^2^, slice thickness 8 mm, inter-slice gap 2 mm, and flip angle 25°. The inversion time (TI) ranged between 260 and 320 ms and was defined based on a separately acquired TI scout. TI was increased by 10 ms for every minute of acquisition to optimally null the signal from the normal myocardium.

### Image analysis and post-processing

Image analysis was performed by a specifically trained reader. For post-processing, a dedicated cardiovascular software (cvi42, V5.11, Circle) was used. Atrial strain analysis was semi-automatically performed as recently described [28] and manually adjusted if necessary.

Volumetric analysis of left atrial (LA) time–volume curves was semi-automatically performed using the biplane area-length calculation technique, which is the method of choice if the Simpsons method is not practical [29]. As the current standard, LA volumes were indexed to body surface area (BSA) [21, 30], then TEF, PEF, and AEF were derived from the time-volume curves according to current literature (Table S2) [27, 31–33].

**Fig. 1:**
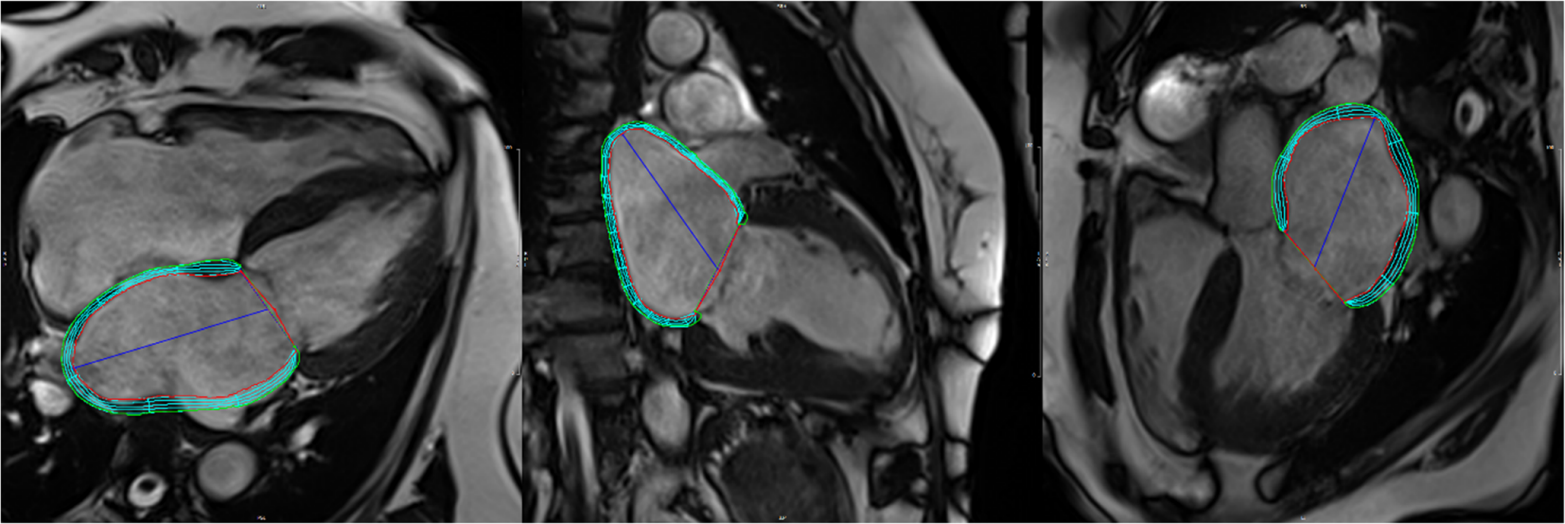
Semiautomatically derived contours of the left atrium in 4-chamber view (left), 2-chamber view (middle), and 3-chamber view (right) in a 69-year-old female AFD patient with enlarged atria

To calculate LA strains, endocardial and epicardial borders were manually traced in a single slice at end-diastole in the 2-, 3-, and 4-chamber-views (Fig. 1). The LA appendage, the pulmonary veins, and the inferior and superior vena cava were excluded, according to standard practice [34–36]. The software subsequently propagated the contours throughout the entire cardiac cycle and thus calculated longitudinal LA strain automatically.

**Fig. 2:**
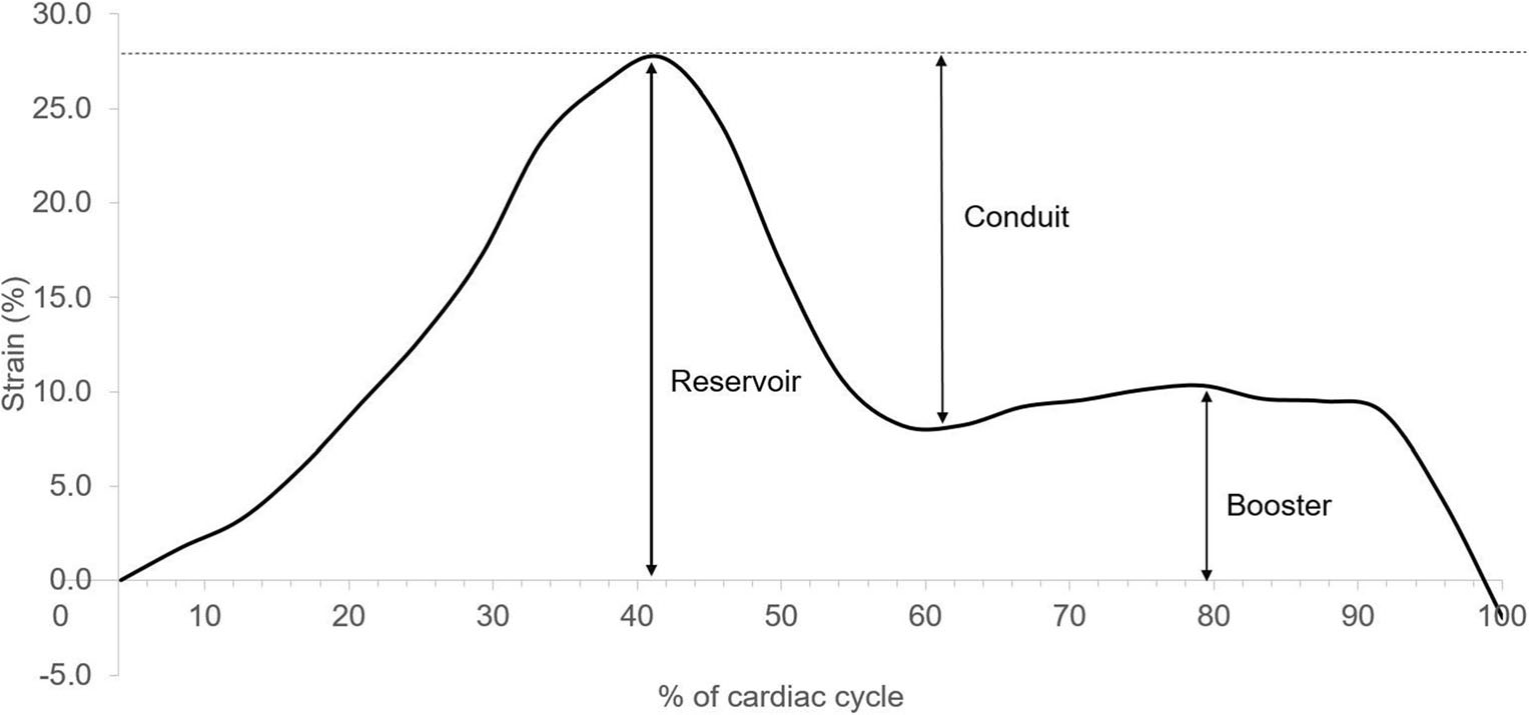
Representative left atrial strain curve with reservoir, conduit, and booster strains

Using the LV end-diastolic frame as a zero-reference, strain curves were then analyzed to identify peaks corresponding to the following phases of the LA cycle: (1) reservoir—passive LA filling due to inflow from pulmonary veins, (2) conduit—passive flow through the LA after the opening of the mitral valve in the early LV filling phase, and (3) booster—active LA contraction during the late LV filling phase [33, 37]. In doing so, reservoir, conduit, and booster strains were derived (Fig. 2).

### Statistical analysis

Statistical analyses were performed using SPSS Statistics (V23, IBM Corp). The Kolmogorov-Smirnov test was used to assess the normal distribution of the continuous data. Continuous variables are reported as means with standard deviations in brackets if normally distributed, and as median and the respective interquartile ranges in case of non-normal distribution. Categorical variables are reported as absolute frequencies and proportions. Differences between AFD phases and HC were compared using two-way ANOVA with post hoc testing and Bonferroni correction. Binary logistic regression models were created to evaluate multiparametric approaches.

To evaluate differences in the ability to discriminate between HC and AFD patients, receiver operating characteristic (ROC) curve analyses were performed and areas under the curve (AUC) were evaluated. Cut-off values were determined by the highest Youden’s index from the comparison between all AFD patients and all HC, as this most closely resembles the real-world scenario where it is not known what disease phase a patient is in ahead of the CMR. These cut-offs were subsequently used for phase-specific evaluation of sensitivity, specificity, and diagnostic accuracy. Pairwise DeLong comparisons were performed to compare different approaches. A *p* value < 0.05 was considered significant.

## Results

### Study population

The overall study population consisted of 58 patients (mean age 40 years [29–51], 31 females) and 58 matched HC (mean age 41 years [25–56], 31 females). Detailed baseline characteristics of the study population can be found in Table 1.

**Table 1:** Baseline characteristics

	Phase I	Matched HC	*p* value
*n* (% female)	19 (53)	19 (53)	1.000
Age, years	32 [14−50]	35 [20−50]	0.526
BMI, kg/m^2^	21.3 [18.4−24.3]	24.6 [18.7−30.5]	**0.041**
BSA, m^2^	1.65 [1.36−1.93]	1.93 [1.66−2.21]	**0.003**
EF, %	61.8 [55.6−67.9]	60.0 [54.5−65.4]	0.337
LVMi, g/m^2^	57.2 [48.4−65.9]	58.2 [49.0−67.4]	0.722
T1, ms	1177 [1114−1241]	1164 [1131−1196]	0.498
LV GLS, %	−15.2 [−19.7 to −10.7]	−18.2 [−21.1 to −15.4]	**0.020**
LA TEF, %	66.8 [57.0−76.6]	65.8 [58.1−73.6]	0.743
LA PEF, %	55.3 [41.8−68.9]	49.8 [38.3−61.2]	0.187
LA AEF, %	38.1 [22.4−53.7]	31.2 [14.6−47.9]	0.207
LA reservoir strain, %	30.7 [19.7−41.7]	46.4 [35.2−57.6]	**< 0.001**
LA conduit strain, %	20.1 [9.5−30.7]	31.1 [23.0−39.1]	**0.001**
LA booster strain, %	12.8 [6.9−18.7]	17.0 [11.2−22.8]	**0.031**
	Phase II	Matched HC	*p* value
*n* (% female)	20 (55)	20 (55)	1.000
Age (years)	39 [27−50]	38 [26−51]	0.929
BMI, kg/m^2^	23.5 [16.8−30.1]	23.6 [21.6−25.7]	0.919
BSA, m^2^	1.86 [1.68−2.04]	1.83 [1.65−2.02]	0.620
EF, %	65.3 [57.4−73.2]	61.8 [56.1−67.4]	0.119
LVMi, g/m^2^	68.9 [52.3−85.6]	58.4 [48.6−68.3]	**0.024**
T1, ms	1117 [1072−1162]	1157 [1105−1208]	**0.022**
LV GLS, %	−15.8 [−19.4 to −12.3]	−20.6 [−23.6 to −17.5]	**< 0.001**
LA TEF, %	64.7 [58−71.4]	67.0 [58−76]	0.369
LA PEF, %	48.6 [38−59.1]	48.5 [36.9−60.1]	0.981
LA AEF, %	40.7 [27.6−53.8]	24.0 [3.8−44.1]	**0.004**
LA reservoir strain, %	29.8 [20.4−39.2]	47.6 [37.4−57.7]	**< 0.001**
LA conduit strain, %	18.0 [10.1−25.9]	31.3 [22.8−39.9]	**< 0.001**
LA booster strain, %	12.8 [8.2−17.4]	17.8 [12.7−22.9]	**0.003**
	Phase III	Matched HC	*p* value
*n* (% female)	19 (53)	19 (53)	1.000
Age (years)	50 [37−63]	50 [37−62]	0.929
BMI, kg/m^2^	25.0 [21.1−28.9]	23.6 [20.2−27.1]	0.247
BSA, m^2^	1.83 [1.64−2.03]	1.86 [1.62−2.10]	0.753
EF, %	64.7 [57.1−72.3]	60.9 [54.9−66.9]	0.092
LVMi, g/m^2^	101.6 [69.6−133.6]	53.3 [46.5−60.2]	**< 0.001**
T1, ms	1089 [1021−1156]	117 [1137−1207]	**< 0.001**
LV GLS, %	−13.6 [−16.1 to −11]	−19.2 [−21.4 to −17]	**< 0.001**
LA TEF, %	52.4 [36.5−68.3]	65.6 [58.2−73.1]	**0.004**
LA PEF, %	36.6 [19.9−53.3]	47.3 [36.9−57.7]	**0.027**
LA AEF, %	33.5 [21.4−45.6]	21.3 [6.1−36.4]	**0.010**
LA reservoir strain, %	21.2 [11.7−30.7]	51.3 [40.8−61.8]	**< 0.001**
LA conduit strain, %	11.6 [5.2−18.0]	33.2 [22.7−43.8]	**< 0.001**
LA booster strain, %	9.9 [5.4−14.3]	20.9 [15.0−26.8]	**< 0.001**

*HC* healthy controls, *BMI* body mass index, *BSA* body surface area, *EF* ejection fraction, *LVMi* left ventricular myocardial mass index, *LV* left ventricular, *GLS* global longitudinal strain, *LA* left atrial, *TEF* total ejection fraction, *PEF* passive ejection fraction, *AEF* active ejection fraction

### Phase I AFD patients

LA strain analyses revealed significant differences between phase I patients and respective matched HC for LA reservoir (30.7% [19.7–41.7] vs. 46.4% [35.2–57.6], *p* < 0.001), LA conduit (20.1% [9.5–30.7] vs. 31.1% [23.0–39.1], p = 0.001), and LA booster strain (12.8% [8.4–15.8] vs. 17.0% [11.2–22.8], *p = *0.031). In addition, LV global longitudinal strain (LV GLS) was significantly reduced (−15.2% [−19.7 to −10.7] vs. −18.2% [−21.1 to −15.4], *p* = 0.020).

LA emptying fractions, LVMi, and global T1 relaxation times did not show significant differences between the groups (all *p* > 0.05). Further details for all parameters can be found in Table 1.

**Fig. 3:**
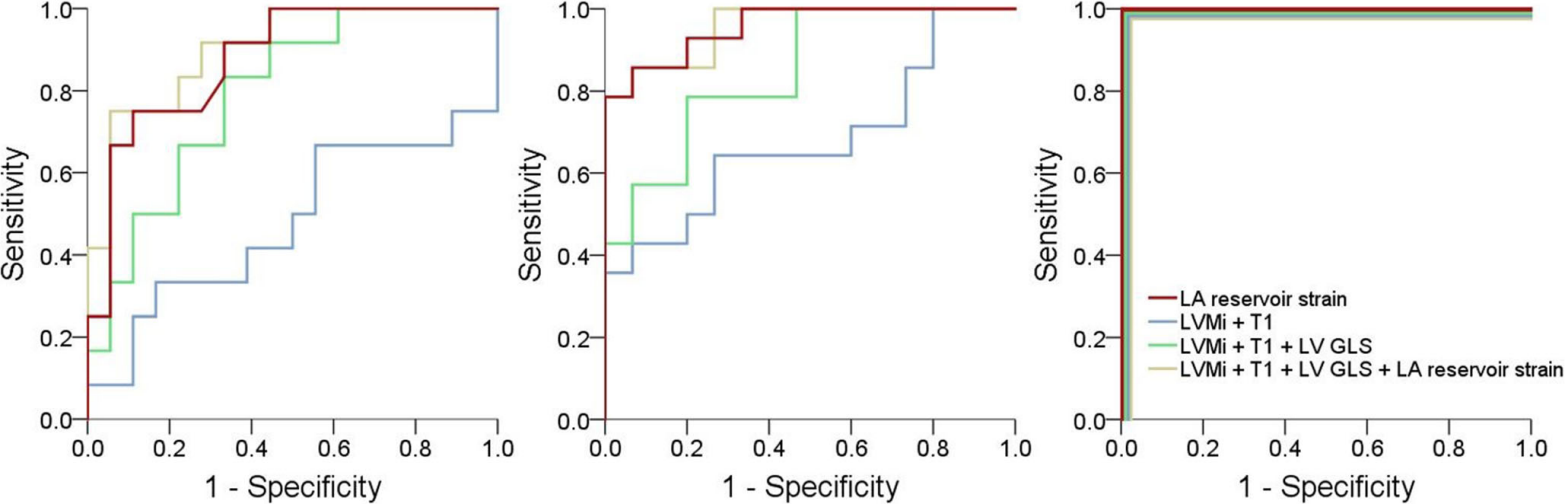
ROC curves for the multiparametric approaches in comparison to LA reservoir strain alone in differentiating HC from AFD patients in phases I-III (left to right), respectively. ROC, receiver operator characteristic; HC, healthy controls; AFD, Anderson-Fabry disease; LA, left atrial; LVMi, Left ventricular mass index

In the ROC-curve analyses, LA reservoir strains reached the highest discriminatory power between phase I patients and matched HC (AUC 0.88). Using the cut-off at 35.9 %, a sensitivity of 89 % and a specificity of 75 % were reached. LVMi (AUC 0.51) and T1 (AUC 0.52) performed significantly lower when compared using the DeLong test (all *p* < 0.02). In multiparametric testing, the addition of LA reservoir strain significantly increased the AUC compared to the combination of LVMi and T1 alone (0.52 vs. 0.90) as well as LVMi, T1, and LV GLS (0.79 vs. 0.90) for differentiating phase I AFD from HC. Comparison between mono- and multiparametric approaches revealed that LA reservoir strain alone was non-inferior to all tested multiparametric approaches (all *p* > 0.211) (Fig. 3). Detailed results for all AUC analyses are reported in Table 2.

**Table 2:** ROC-curve analyses

All AFD patients vs. all HC	AUC	Cut-off	Sensitivity	Specificity
LA reservoir strain	0.96	35.9 %	94 %	85 %
LA conduit strain	0.93	22.9 %	92 %	88 %
LA booster strain	0.84	12.4 %	90 %	63 %
LVMi	0.76	63.3 g/m^2^	79 %	68 %
T1	0.72	1132 ms	89 %	61 %
LV GLS	0.87	-15.7 %	94 %	76 %
LVMi+T1*	0.77	0.44	68 %	87 %
LVMi+T1+LV GLS^ꝉ^	0.89	0.33	88 %	75 %
LVMi+T1+LV strain+LA strain^Ŧ^	0.96	0.36	93 %	89 %
Phase I vs. matched HC	AUC	Sensitivity	Specificity	Accuracy
LA reservoir strain	0.88	89 %	75 %	82 %
LA conduit strain	0.86	89 %	83 %	86 %
LA booster strain	0.75	78 %	67 %	73 %
LVMi	0.51	72 %	42 %	57 %
T1	0.52	89 %	33 %	61 %
LV GLS	0.82	89 %	75 %	82 %
LVMi+T1*	0.52	33 %	78 %	56 %
LVMi+T1+LV GLS^ꝉ^	0.79	83 %	67 %	75 %
LVMi+T1+LV strain+LA strain^Ŧ^	0.90	92 %	72 %	82 %
Phase II vs. matched HC	AUC	Sensitivity	Specificity	Accuracy
LA reservoir strain	0.96	93 %	79 %	86 %
LA conduit strain	0.91	87 %	79 %	83 %
LA booster strain	0.80	87 %	50 %	69 %
LVMi	0.67	67 %	57 %	62 %
T1	0.76	80 %	64 %	72 %
LV GLS	0.85	93 %	64 %	79 %
LVMi+T1*	0.68	64 %	73 %	69 %
LVMi+T1+LV GLS^ꝉ^	0.85	79 %	80 %	80 %
LVMi+T1+LV strain+LA strain^Ŧ^	0.96	86 %	93 %	90 %
Phase III vs. matched HC	AUC	Sensitivity	Specificity	Accuracy
LA reservoir strain	1.00	100 %	100 %	100 %
LA conduit strain	1.00	95 %	100 %	98 %
LA booster strain	0.97	100 %	67 %	84 %
LVMi	0.99	95 %	93 %	94 %
T1	0.85	95 %	80 %	88 %
LV GLS	0.94	100 %	80%	90 %
LVMi+T1*	1.00	100 %	100 %	100 %
LVMi+T1+LV GLS^ꝉ^	1.00	100 %	84 %	92 %
LVMi+T1+LV strain+LA strain^Ŧ^	1.00	100 %	100 %	100 %

*ROC* receiver operating characteristic, *HC* healthy controls, *AUC* area under the curve, *LA* left atrial, *LVMi* left ventricular myocardial mass index, *LV GLS* left ventricular global longitudinal strain

*regression equation: −0.27 − 0.004(*T*1) + 0.07(*LVMi*)

^ꝉ^ regression equation: 3.15 − 0.05(*LVMi*) + 0.42(*LV GLS*)

^Ŧ^regression equation: 12.39 − 0.001(*T*1) + 0.01(*LVMi*) + 0.22(*LV GLS*) − 0.30(*LA reservoir strain*)

### Phase II AFD patients

**Fig. 4:**
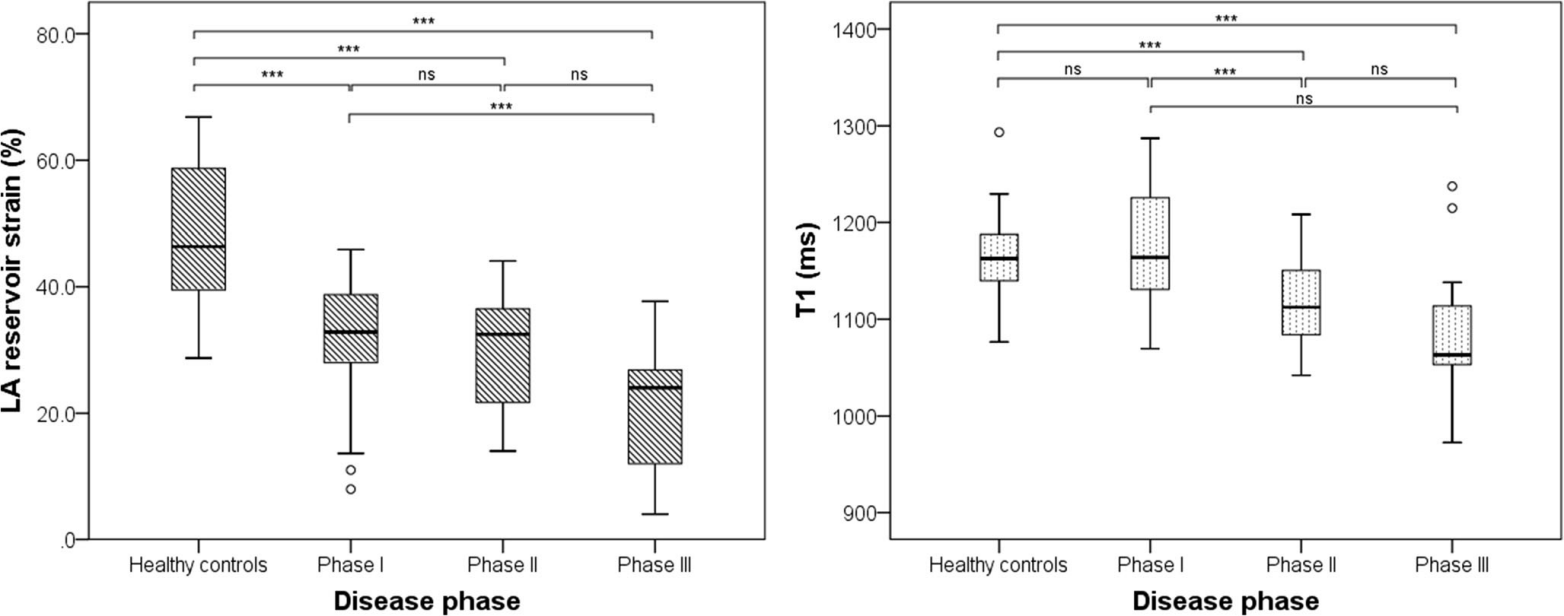
Boxplots showing a gradual reduction in LA reservoir strain (left) and T1 relaxation times (right) over all three AFD phases. Significant differences between groups are marked with brackets and asterisks. LA, left atrial; ns, not significant

As seen in Fig. 4, mean T1 relaxation times were significantly shortened in phase II in comparison to HC (*p* = 0.022) and phase I patients (*p* = 0.012). LVMi was significantly increased in comparison to matched HC (68.9 g/m^2^ [52.3–85.6] vs. 58.4 g/m^2^ [48.6–68.3], *p* = 0.024) but not in comparison to phase I patients (57.2 g/m^2^ [48.4–65.9], *p* = 0.165). In ROC-curve analyses, T1 showed an AUC of 0.76 (sensitivity 80 %, specificity 64 %) and LVMi reached an AUC of 0.67 (sensitivity 67 % and specificity 57 %).

LA strain analysis revealed significant differences between phase II AFD and respective matched HC for reservoir (29.8% [20.4–39.2] vs. 47.6% [37.4–57.7], *p* < 0.001), conduit (18.0% [10.1–25.9] vs. 31.3% [22.8–39.9], *p* < 0.001), and booster strain (12.8 [8.2–17.4] vs. 17.8 [12.7–22.9], *p* = 0.003). In addition, LV global longitudinal strain (LV GLS) was significantly reduced in comparison to HC (−15.8% [−19.4 to −12.3] vs. −20.6% [−23.6 to −17.5], *p* < 0.001) but not in comparison to phase I AFD patients (*p* = 1.000). ROC-curve analyses indicated that LA reservoir strain (AUC 0.96, sensitivity 89 %, and specificity 75 %) performed superior to LVMi and T1 (*p* = 0.024 and 0.023, respectively).

The comparison between multiparametric approaches showed a significant increase of the AUC when LA reservoir strain was added to the combination of LVMi and T1 (AUC 0.68 vs. 0.96) as well as LVMi, T1, and LV GLS (0.79 vs. 0.96). Comparison between mono- and multiparametric approaches revealed that LA reservoir strain alone was superior to the combination of LVMi and T1 as well as non-inferior to all tested multiparametric approaches (all *p* ≥ 0.088) (Fig. 3). Concerning the functional volumetric measurements, only AEF was significantly higher in phase II AFD patients compared to HC (40.7% [27.6–53.8] vs. 24.0% [3.8–44.1], *p* = 0.004).

### Phase III AFD patients

With the progression of AFD, established parameters such as LVMi and myocardial T1 gain more importance. Thus, LVMi was significantly increased in phase III AFD compared to HC (101.6 g/m^2^ [69.6–133.6] vs. 53.3 g/m^2^ [46.5–60.2], *p* < 0.001), to phase I (57.2 g/m^2^ [48.4–65.9], *p* < 0.001), and to phase II (68.9 g/m^2^ [52.3–85.6], *p* < 0.001) patients. Figure 4 shows significantly shortened T1 relaxation times compared to HC and phase I (*p* < 0.001); however, there were no significant differences between phases II and III (*p* = 0.706).

In ROC-curve analyses, T1 reached an AUC of 0.85 (sensitivity 95 % and specificity 80 %) for the differentiation between phase III patients and HC, while LVMi reached an AUC of 0.99 (sensitivity 95 % and specificity 93 %).

All LA strain parameters showed significant differences between phase III patients and matched HC (all *p* < 0.001) (Fig. 4). Again, LV global longitudinal strain (LV GLS) was significantly reduced in comparison to HC (−13.6% [−16.1 to −11.0] vs. −19.2% [−21.4 to −17.0], *p* < 0.001) but not in comparison to the other disease phases (all *p* ≥ 0.202). ROC-curve analyses revealed non-inferior diagnostic performance of LA reservoir strain (AUC 1.00, sensitivity 100 %, and specificity 100 %) in comparison to LVMi (AUC 0.96, sensitivity 95 %, and specificity 93 %, *p* = 0.408) and T1 (AUC 0.85, sensitivity 95 %, and specificity 80 %, *p* = 0.0.088) and LV GLS (AUC 0.94, sensitivity 100 %, and specificity 80 %, *p* = 0.124). All multiparametric approaches reached remarkably high diagnostic confidence (AUC 1.00).

### Sex-specific results

As AFD is an x-linked disease, results must also be viewed as split by sex. Comparisons between male and female patients showed a significantly smaller LA TEF for phase I male patients, significantly younger male patients in phases II and III, and a significantly higher increase in LVMi for male patients in both phases II and III. Significantly lower (yet still normal) LV EF was found in male phase II patients and significantly higher LA PEF was observed in male phase III patients. The detailed comparison of sex-specific baseline characteristics for AFD patients can be found in Table 3.

**Table 3:** Comparison between female and male AFD patients

Phase I	Female	Male	*p* value
*n*	10	9	
Age, years	36 [21–50]	29 [6–49]	0.376
BMI, kg/m^2^	21.3 [18.6–23.9]	21.3 [17.9–24.8]	0.964
BSA, m^2^	1.6 [1.3–1.8]	1.7 [1.4–2]	0.370
EF, %	63.9 [57.9–69.9]	59.5 [53.7–65.3]	0.124
LVMi, g/m^2^	55.1 [47.5–62.7]	59.4 [49.6–69.2]	0.302
T1, ms	1205 [1141–1269]	1150 [1095–1205]	0.139
LV GLS, %	–17.7 [–21.9 to –13.5]	–15.5 [–19.1 to –11.9]	**0.019**
LA TEF, %	72.5 [64.2–80.8]	61.0 [53.2–68.9]	**0.008**
LA PEF, %	57.6 [48.3–66.8]	53.1 [36–70.2]	0.506
LA AEF, %	40.6 [23.5–57.7]	35.5 [20.8–50.3]	0.509
LA reservoir strain, %	34.1 [23.1–45]	27 [16.6–37.4]	0.165
LA conduit strain, %	20.1 [9.3–30.9]	20.1 [9.2–31]	0.998
LA booster strain, %	15.1 [8.2–22]	10.2 [7–13.4]	0.063
Phase II	Female	Male	*p* value
*n*	11	9	
Age (years)	46 [39–54]	30 [21–38]	**< 0.001**
BMI, kg/m^2^	25.4 [16.8–34]	21.4 [18.6–24.1]	0.197
BSA, m^2^	1.9 [1.7–2.1]	1.8 [1.7–2]	0.462
EF, %	70.6 [64.3–76.9]	59.4 [54.8–64]	**< 0.001**
LVMi, g/m^2^	57.5 [46.1–68.9]	81.7 [70.1–93.2]	**< 0.001**
T1, ms	1120 [1087–1153]	1113 [1055–1171]	0.777
LV GLS, %	–19.6 [–23.3 to –15.8]	–15.6 [–19 to –12.2]	**< 0.001**
LA TEF, %	63.9 [57–70.7]	65.8 [59–72.6]	0.534
LA PEF, %	44.7 [38.2–51.3]	53.2 [40.4–66.1]	0.099
LA AEF, %	38.6 [24.2–52.9]	43.3 [31.6–55]	0.430
LA reservoir strain, %	30.6 [20.6–40.5]	28.9 [19.8–38]	0.695
LA conduit strain, %	17.9 [9.6–26.1]	18.2 [10.3–26.2]	0.920
LA booster strain, %	13.2 [8.4–18]	12.3 [7.7–16.8]	0.651
Phase III	Female	Male	*p* value
*n*	10	9	
Age (years)	57 [47–68]	42 [32–53]	**0.008**
BMI, kg/m^2^	26 [21.5–30.6]	23.9 [21.1–26.8]	0.248
BSA, m^2^	1.8 [1.6–2.1]	1.8 [1.7–1.9]	0.995
EF, %	63.9 [54.3–73.5]	65.6 [60.6–70.6]	0.632
LVMi, g/m^2^	85.5 [60.4–110.5]	119.5 [89.3–149.7]	**0.018**
T1, ms	1107 [1046–1168]	1068 [995–1141]	0.289
LV GLS, %	–16.8 [–21.3 to –12.2]	–16.0 [–18.6 to –13.4]	**< 0.001**
LA TEF, %	45.3 [27.4–63.2]	59.5 [49.4–69.6]	0.059
LA PEF, %	27.2 [14.9–39.6]	45.9 [30.2–61.6]	**0.013**
LA AEF, %	30.9 [15.2–46.7]	36.1 [29.2–43]	0.383
LA reservoir strain, %	18.2 [7.2–29.3]	24.5 [18–31.1]	0.147
LA conduit strain, %	9.8 [3.1–16.5]	13.6 [7.9–19.3]	0.203
LA booster strain, %	8.5 [3.5–13.4]	11.4 [8–14.8]	0.145

*HC* healthy controls, *BMI* body mass index, *BSA* body surface area, *EF* ejection fraction, *LVMi* left ventricular myocardial mass index, *LV* left ventricular, *GLS* global longitudinal strain, *LA* left atrial, *TEF* total ejection fraction, *PEF* passive ejection fraction, *AEF* active ejection fraction

In addition, Table 4 shows detailed sex-specific cut-off points and diagnostic accuracies for the comparison of all AFD patients with all HC. In short, LA reservoir and conduit strains as well as LV GLS showed only small differences in optimal cut-offs between women and men (< 1 %), while other parameters had slightly higher sex-specific differences.

**Table 4:** Sex-specific cut-offs from ROC-curve analyses

All AFD patients vs. all HC	Female			Male		
	AUC	Cut-off	sens/spec	AUC	cut-off	Sens/spec
LA reservoir strain	0.96	36.2 %	97/81 %	0.96	35.5 %	96/ 95 %
LA conduit strain	0.94	22.5 %	93/ 86 %	0.91	23.0 %	91/ 90 %
LA booster strain	0.87	12.3 %	97/ 67 %	0.81	16.3 %	64/ 95 %
LVMi	0.76	62.4 g/m^2^	93/ 62 %	0.80	69.9 g/m^2^	82/ 75 %
T1	0.75	1147 ms	90/ 62 %	0.71	1132 ms	82/ 70%
LV GLS	0.93	15.7 %	100/ 71 %	0.82	15.8 %	86/ 80%
LVMi+T1*	0.78	0.51	67/ 93 %	0.80	0.44	75/ 86 %
LVMi+T1+LV GLS^ꝉ^	0.96	0.23	100/ 83 %	0.90	0.28	90/ 77 %
LVMi+T1+LV strain+LA strain^Ŧ^	0.98	0.24	100/ 90 %	0.98	0.31	100/ 96 %

*ROC* receiver operating characteristic, *HC* healthy controls, *AUC* area under the curve, *LA* left atrial, *LVMi* left ventricular myocardial mass index, *LV GLS* left ventricular global longitudinal strain

*regression equations:

- female 3.74 − 0.008(*T*1) + 0.10(*LVMi*)

- male  − 8.73 − 0.001(*T*1) + 0.10(*LVMi*)

^ꝉ^ regression equations:

- female 18.53 − 0.009(*T*1) + 0.13(*LVMi*) + 0.87(*LV GLS*)

- male −3.76 + 0.03(*T*1) + 0.09(*LVMi*) + 0.37(*LV GLS*)

^Ŧ^regression equations:

- female 35.23 − 0.012(*T*1) + 0.09(*LVMi*) + 0.81(*LV GLS*) − 0.28(*LA reservoir strain*)

- male 52.74 − 0.019(*T*1) − 0.03(*LVMi*) + 0.53(*LV GLS*) − 0.61(*LA reservoir strain*)

### Correlation of LA strain and functional parameters with disease severity

Significant positive correlations were observed between LA reservoir strain and T1 (Pearson’s *r* = 0.40 (female) and *r* = 0.28 (male), *p* ≤ 0.05). In addition, there were negative correlations between LA reservoir strain and LVMi for both women *(r* = −0.47, *p* < 0.001) and men (*r* = 0.42, *p* = 0.001) (Fig. S4).

## Discussion

In this study, various LA parameters were analyzed in a multi-parametric CMR diagnostic workup of AFD patients. Disease severity and progression were evaluated based on novel atrial, as well as already established parameters, such as functional markers, LV strain, and myocardial T1. The results demonstrated that LA reservoir strain reliably differentiates between early-phase AFD patients and HC when atrial volumetric parameters and T1 mapping fail to do so. In addition, this study found a significant correlation between LA strains and disease severity in AFD.

Previous studies have shown that abnormalities in myocardial T1 occur before LV hypertrophy develops [10–12, 38]. Therefore, T1 mapping complements myocardial volumetry and LGE imaging for the detection of cardiac involvement and the monitoring of disease progression. While the diagnosis and treatment of classic AFD have become standardized and straightforward, certain patients, such as women and those with rarer gene mutations may experience a challenging path to establish their diagnosis. This is due to the X-linked inheritance pattern, reduced penetrance, and variable expression [39, 40]. Therefore, it remains crucial to evaluate new parameters for their potential to detect myocardial involvement as early as possible.

The single CMR-based atrial strain study available in the literature was designed to compare AFD patients with severe LV hypertrophy (equivalent to phase III patients in this study) and patients with hypertrophic cardiomyopathy and thus had a different scope [14]. However, to the extent a comparison is warranted, the ranges of LA reservoir strain in phase III AFD patients from this study were within one standard deviation of the above-mentioned study population (21.2% [11.7–30.7] in this study vs. 25% [19–31] in Moroni et al [14]).

### LA volumetry

An STE-based study has shown that abnormalities of LA functional parameters occur in AFD patients before LV hypertrophy becomes evident [13]. Interestingly, in this study, LA volumetric parameters had no diagnostic impact in the early disease phases. LA emptying fractions (TEF, PEF, and AEF) showed pathologically altered values only in patients with advanced disease (phase III). This is most likely due to the fact that impairment of deformation patterns, therefore abnormalities in strain, do not implicate functional impairment in the early phases.

In addition, this study confirms prior literature findings that AEF proved to be the least diagnostically accurate in comparison to TEF and PEF [41, 42]. This may be explained by the nature of this parameter and its respective phase of the cardiac cycle, as small errors in contouring have a higher impact on parameters that rely on the detailed acquisition of small values.

### LA strain analysis

Prior research demonstrated that ventricular strain analysis by CMR could show AFD-related strain abnormalities even before sphingolipid deposition can be detected by mapping sequences [16]. The results corroborate these findings as LA strain parameters were able to confidently differentiate phenotypically negative AFD patients from HC. Phase I AFD patients showed similar impairment of atrial deformation patterns as patients with abnormal myocardial T1 (phase II). In addition, LA reservoir strain correlated with disease severity and allowed for the stratification of AFD patients with advanced disease (phases II and III). In contrast, T1 is better suited for the detection of early disease, while LVMi is more useful in advanced AFD. While T1 relaxation times are abnormally short in early disease (phase II), they can pseudo-normalize in the presence of myocardial fibrosis (phase III), therefore reducing diagnostic confidence. To an extent, this can also be seen in this study’s data, as there were no significant differences in T1 relaxation times between phases II and III, and diagnostic confidence from T1 mapping alone was inferior to LVMi in phase III patients. LVMi, on the other hand, can be mostly seen pathologically increased in advanced disease and is not able to confidently detect early disease (phases I and II). As previously reported, LA booster strain had the lowest diagnostic performance among atrial strain parameters [21, 43, 44]. Importantly, ROC analyses also proved that atrial strain assessment has an incremental value when compared to multiparametric approaches including ventricular strains.

From a pathophysiological viewpoint, the early impairment and gradual deterioration of LA strain with advancing disease could be explained by an accumulation of multiple pathways with abnormal atrial deformation patterns as their common outcome [45, 46]:

1. Deposition of sphingolipids in the atrial myocardium

Histopathological studies have proven that sphingolipid deposition does occur in the atria [47]. Unfortunately, as the atrial myocardium is extremely thin, T1 mapping cannot be performed in order to correlate LA reservoir strain with the amount of fatty deposition. Atrial hypertrophy is not commonly found in AFD patients, which sets AFD apart from other storage diseases such as amyloidosis and makes primary atrial involvement preceding ventricular involvement less likely to be the main cause for the findings.

(2) Ventricular diastolic dysfunction

The strain has recently been highlighted in diseases accompanied by diastolic dysfunction [26, 30, 48–50]. In addition, it has been shown that myocardial lipid depositions can lead to diastolic dysfunction which ultimately progresses into restrictive cardiomyopathy [51]. The results of this study suggest that this mechanism may occur at earlier disease phases than previously thought. Corroborating this thesis is that LA reservoir strain was the most accurate predictor of cardiac involvement in this study. Its nature and corresponding phase of the cardiac cycle make it a parameter both reliant on atrial mechanics itself, as well as the mitral annular plane excursion, and therefore suggests high sensitivity for diastolic dysfunction [52].

Overall, this study complements the current literature in regard to atrial strain analysis and involvement in AFD by demonstrating its diagnostic performance. It provides novel insights into atrial deformation in AFD patients, suggesting latent diastolic dysfunction pre-detectable accumulation. In addition, it was able to show a correlation between disease severity and LA reservoir strain. The careful evaluation of the atria could aid in further understanding the different pathophysiological pathways in which the disease impairs cardiac function and potentially help guide therapeutic decisions in the future [53].

## Limitations

This study has several limitations. Despite the balanced sex ratio and efforts to manage an equal distribution of HC and patients with AFD, this remains a single-center, retrospective study. Several studies have already validated the reproducibility of atrial strain analyses; however, there are still significant differences between various vendors. Thus, there exists no current reference standard [26, 28, 54]. Due to its retrospective nature, this study cannot provide outcome data. Further studies are needed to investigate the connection between pathological atrial strain values on disease progression. Only with that knowledge, the benefits of starting ERT at the earliest time possible can be weighed against the challenges of a lifelong bi-weekly infusion regimen with its innate risk of immune responses limiting later use of the ERT [55].

Because of the retrospective nature of the study, patients did not undergo endomyocardial biopsy as ground truth for cardiac involvement in AFD. However, as a surrogate, thorough genetic analyses were performed, and all mutations with their respective nucleotides and protein changes are reported in Table S3.

## Conclusion

The analysis of atrial deformation patterns by CMR feature-tracking strain analysis revealed that LA reservoir strain reliably differentiates between early-phase AFD patients and HC whereas atrial volumetric parameters and T1 mapping fail to do so. In addition, atrial strain analysis provides incremental value in the diagnosis of early AFD when compared to multiparametric approaches including ventricular strain analysis. This study also found a significant correlation between LA strains and disease severity in AFD measured by T1 and LVMi. These results suggest that the evaluation of LA deformation patterns should be an integral part of the diagnostic work-up in AFD with questionable cardiac involvement. Further studies are needed to evaluate whether earlier initiation of ERT based on these results can help minimize cardiac complications from AFD.

## Supplementary information

ESM 1 (DOCX 154 kb)

## Abbreviations

AEF: Active atrial emptying fraction
AFD: Anderson-Fabry disease
CMR: Cardiac magnetic resonance imaging
ECG: Electrocardiogram
ERT: Enzyme replacement therapy
FOV: Field of view
GLS: Global longitudinal strain
HC: Healthy controls
LA: Left atrial
LGE: Late gadolinium enhancement
LV: Left ventricular
LVMi: Left ventricular mass index
PEF: Passive atrial emptying fraction
ROC: Receiver operating characteristic
STE: Speckle tracking echocardiography
TEF: Total atrial emptying fraction

## Declarations

### Statistics and biometry

No complex statistical methods were necessary for this paper.

### Study subjects or cohorts overlap

Some study subjects or cohorts have been previously reported in studies concerning ventricular strain parameters, which are currently under review. This study contains parts of the doctoral thesis of one of theauthors (C.R.)

### Methodology

• retrospective

• observational

• performed at one institution

## References

[1] Desnick RJBD, Scriver C, Beaudet A, Sly W, Valle D (1989). Fabry disease: α-galactosidase A deficiency; Schindler disease: α-N-acetylgalactosaminidase deficiency. The metabolic basis of inherited disease.

[2] Mehta A HD. Fabry Disease. In: Adam MP, Ardinger HH, Pagon RA, et al, eds. GeneReviews® Seattle University of Washington, 2002.

[3] Baig S, Edward NC, Kotecha D, Liu B, Nordin S, Kozor R (2018). Ventricular arrhythmia and sudden cardiac death in Fabry disease: a systematic review of risk factors in clinical practice. Europace.

[4] Nordin S, Kozor R, Medina-Menacho K, Abdel-Gadir A, Baig S, Sado DM (2019). Proposed stages of myocardial phenotype development in Fabry disease. JACC Cardiovasc Imaging.

[5] Eng CM, Guffon N, Wilcox WR, Germain DP, Lee P, Waldek S (2001). Safety and efficacy of recombinant human alpha-galactosidase A replacement therapy in Fabry's disease. N Engl J Med.

[6] Weidemann F, Niemann M, Breunig F, Herrmann S, Beer M, Stork S (2009). Long-term effects of enzyme replacement therapy on fabry cardiomyopathy: evidence for a better outcome with early treatment. Circulation..

[7] El Dib R, Gomaa H, Carvalho RP, Camargo SE, Bazan R, Barretti P (2016). Enzyme replacement therapy for Anderson-Fabry disease. Cochrane Database Syst Rev.

[8] Goker-Alpan O, Longo N, McDonald M, Shankar SP, Schiffmann R, Chang P, et al. An open-label clinical trial of agalsidase alfa enzyme replacement therapy in children with Fabry disease who are naive to enzyme replacement therapy. Drug Des Devel Ther 2016;10:1771-1781. 10.2147/DDDT.S102761 PMC4887054 27307708

[9] Tsuboi K, Yamamoto H (2017). Efficacy and safety of enzyme-replacement-therapy with agalsidase alfa in 36 treatment-naive Fabry disease patients. BMC Pharmacol Toxicol.

[10] Moon JC, Sachdev B, Elkington AG, McKenna WJ, Mehta A, Pennell DJ (2003). Gadolinium enhanced cardiovascular magnetic resonance in Anderson-Fabry disease. Evidence for a disease specific abnormality of the myocardial interstitium. Eur Heart J.

[11] Moon JC, Sheppard M, Reed E, Lee P, Elliott PM, Pennell DJ (2006). The histological basis of late gadolinium enhancement cardiovascular magnetic resonance in a patient with Anderson-Fabry disease. J Cardiovasc Magn Reson.

[12] Augusto JB, Johner N, Shah D, Nordin S, Knott KD, Rosmini S et al (2020) The myocardial phenotype of Fabry disease pre-hypertrophy and pre-detectable storage. Eur Heart J Cardiovasc Imaging 10.1093/ehjci/jeaa101 PMC8219366 32514567

[13] Boyd AC, Lo Q, Devine K, Tchan MC, Sillence DO (2013). Sadick N, et al Left atrial enlargement and reduced atrial compliance occurs early in Fabry cardiomyopathy. J Am Soc Echocardiogr.

[14] Moroni A, Tondi L, Camporeale A, Milani V, Pica S, Pieroni M (2021). Left atrial morpho-functional changes in hypertrophic cardiomyopathy and Fabry disease: a CMR-feature tracking study. Eur Heart J Cardiovasc Imaging.

[15] Pichette M, Serri K, Page M, Di LZ, Bichet DG, Poulin F (2017). Impaired left atrial function in fabry disease: a longitudinal speckle-tracking echocardiography study. J Am Soc Echocardiogr.

[16] Vijapurapu R, Nordin S, Baig S, Liu B, Rosmini S, Augusto J (2019). Global longitudinal strain, myocardial storage and hypertrophy in Fabry disease. Heart..

[17] Kuppahally SS, Akoum N, Burgon NS, Badger TJ, Kholmovski EG, Vijayakumar S (2010). Left atrial strain and strain rate in patients with paroxysmal and persistent atrial fibrillation: relationship to left atrial structural remodeling detected by delayed-enhancement MRI. Circ Cardiovasc Imaging.

[18] Evin M, Cluzel P, Lamy J, Rosenbaum D, Kusmia S, Defrance C (2015). Assessment of left atrial function by MRI myocardial feature tracking. J Magn Reson Imaging.

[19] Kebed KY, Addetia K, Lang RM (2019). Importance of the Left Atrium: More Than a Bystander?. Heart Fail Clin.

[20] Latif SR, Nguyen VQ, Peters DC, Soufer A, Henry ML, Grunseich K (2019). Left atrial fibrosis correlates with extent of left ventricular myocardial delayed enhancement and left ventricular strain in hypertrophic cardiomyopathy. Int J Card Imaging.

[21] Schuster A, Backhaus SJ, Stiermaier T, Navarra JL, Uhlig J, Rommel KP (2019). Left atrial function with MRI enables prediction of cardiovascular events after myocardial infarction: insights from the AIDA STEMI and TATORT NSTEMI trials. Radiology..

[22] Tops LF, Schalij MJ, Bax JJ (2010). Imaging and atrial fibrillation: the role of multimodality imaging in patient evaluation and management of atrial fibrillation. Eur Heart J.

[23] Agner BF, Kuhl JT, Linde JJ, Kofoed KF, Akeson P (2014). Rasmussen BV, et al Assessment of left atrial volume and function in patients with permanent atrial fibrillation: comparison of cardiac magnetic resonance imaging, 320-slice multi-detector computed tomography, and transthoracic echocardiography. Eur Heart J Cardiovasc Imaging.

[24] Kuchynka P, Podzimkova J, Masek M, Lambert L, Cerny V, Danek B (2015). The Role of magnetic resonance imaging and cardiac computed tomography in the assessment of left atrial anatomy, size, and function. Biomed Res Int.

[25] Ponikowski P, Voors AA, Anker SD, Bueno H, Cleland JGF, Coats AJS (2016). 2016 ESC Guidelines for the diagnosis and treatment of acute and chronic heart failure: the Task Force for the diagnosis and treatment of acute and chronic heart failure of the European Society of Cardiology (ESC)Developed with the special contribution of the Heart Failure Association (HFA) of the ESC. Eur Heart J.

[26] Lamy J, Soulat G, Evin M, Huber A, de Cesare A, Giron A (2018). Scan-rescan reproducibility of ventricular and atrial MRI feature tracking strain. Comput Biol Med.

[27] Blume GG, McLeod CJ, Barnes ME, Seward JB, Pellikka PA, Bastiansen PM (2011). Left atrial function: physiology, assessment, and clinical implications. Eur J Echocardiogr.

[28] Truong VT, Palmer C, Wolking S, Sheets B, Young M, Ngo TNM (2020). Normal left atrial strain and strain rate using cardiac magnetic resonance feature tracking in healthy volunteers. Eur Heart J Cardiovasc Imaging.

[29] Nacif MS, Barranhas AD, Turkbey E, Marchiori E, Kawel N (2013). Mello RA, et al Left atrial volume quantification using cardiac MRI in atrial fibrillation: comparison of the Simpson's method with biplane area-length, ellipse, and three-dimensional methods. Diagn Interv Radiol.

[30] Leng S, Ge H, He J, Kong L, Yang Y, Yan F (2020). Long-term prognostic value of cardiac MRI left atrial strain in ST-segment elevation myocardial infarction. Radiology..

[31] Maceira AM, Cosin-Sales J, Prasad SK, Pennell DJ (2016). Characterization of left and right atrial function in healthy volunteers by cardiovascular magnetic resonance. J Cardiovasc Magn Reson.

[32] Altmann S, Halfmann MC, Abidoye I, Yacoub B, Schmidt M, Wenzel P (2021). Compressed sensing acceleration of cardiac cine imaging allows reliable and reproducible assessment of volumetric and functional parameters of the left and right atrium. Eur Radiol.

[33] Hoit BD (2014). Left atrial size and function: role in prognosis. J Am Coll Cardiol.

[34] Maceira AM, Cosin-Sales J, Roughton M, Prasad SK, Pennell DJ (2010). Reference left atrial dimensions and volumes by steady state free precession cardiovascular magnetic resonance. J Cardiovasc Magn Reson.

[35] Maceira AM, Cosin-Sales J, Roughton M, Prasad SK, Pennell DJ (2013). Reference right atrial dimensions and volume estimation by steady state free precession cardiovascular magnetic resonance. J Cardiovasc Magn Reson.

[36] Le Ven F, Bibeau K, De Larochelliere E, Tizon-Marcos H, Deneault-Bissonnette S, Pibarot P (2016). Cardiac morphology and function reference values derived from a large subset of healthy young Caucasian adults by magnetic resonance imaging. Eur Heart J Cardiovasc Imaging.

[37] To AC, Flamm SD, Marwick TH, Klein AL (2011). Clinical utility of multimodality LA imaging: assessment of size, function, and structure. JACC Cardiovasc Imaging.

[38] Sado DM, White SK, Piechnik SK, Banypersad SM, Treibel T, Captur G (2013). Identification and assessment of Anderson-Fabry disease by cardiovascular magnetic resonance noncontrast myocardial T1 mapping. Circ Cardiovasc Imaging.

[39] Schiffmann R, Fuller M, Clarke LA, Aerts JM (2016). Is it Fabry disease?. Genet Med.

[40] Vardarli I, Rischpler C, Herrmann K, Weidemann F (2020). Diagnosis and screening of patients with fabry disease. Ther Clin Risk Manag.

[41] Chirinos JA, Sardana M, Ansari B, Satija V, Kuriakose D, Edelstein I (2018). Left atrial phasic function by cardiac magnetic resonance feature tracking is a strong predictor of incident cardiovascular events. Circ Cardiovasc Imaging.

[42] Yang Y, Yin G, Jiang Y, Song L, Zhao S, Lu M (2020). Quantification of left atrial function in patients with non-obstructive hypertrophic cardiomyopathy by cardiovascular magnetic resonance feature tracking imaging: a feasibility and reproducibility study. J Cardiovasc Magn Reson.

[43] von Roeder M, Rommel KP, Kowallick JT, Blazek S, Besler C, Fengler K (2017). Influence of left atrial function on exercise capacity and left ventricular function in patients with heart failure and preserved ejection fraction. Circ Cardiovasc Imaging.

[44] Leng S, Dong Y, Wu Y, Zhao X, Ruan W (2019). Zhang G, et al Impaired cardiovascular magnetic resonance-derived rapid semiautomated right atrial longitudinal strain is associated with decompensated hemodynamics in pulmonary arterial hypertension. Circ Cardiovasc Imaging.

[45] Ferrans VJ, Hibbs RG, Burda CD (1969). The heart in Fabry's disease. A histochemical and electron microscopic study. Am J Cardiol.

[46] Kampmann C, Baehner F, Ries M, Beck M (2002). Cardiac involvement in Anderson-Fabry disease. J Am Soc Nephrol.

[47] Sheppard MN, Cane P, Florio R, Kavantzas N, Close L, Shah J (2010). A detailed pathologic examination of heart tissue from three older patients with Anderson-Fabry disease on enzyme replacement therapy. Cardiovasc Pathol.

[48] Tops LF, van der Wall EE, Schalij MJ, Bax JJ (2007). Multi-modality imaging to assess left atrial size, anatomy and function. Heart..

[49] Huber AT, Lamy J, Rahhal A, Evin M, Atassi F, Defrance C (2018). Cardiac MR Strain: a noninvasive biomarker of fibrofatty remodeling of the left atrial myocardium. Radiology..

[50] Pieske B, Tschöpe C, de Boer RA, Fraser AG, Anker SD, Donal E (2019). How to diagnose heart failure with preserved ejection fraction: the HFA–PEFF diagnostic algorithm: a consensus recommendation from the Heart Failure Association (HFA) of the European Society of Cardiology (ESC). Eur Heart J.

[51] Linhart A, Palecek T, Bultas J, Ferguson JJ, Hrudova J, Karetova D (2000). New insights in cardiac structural changes in patients with Fabry’s disease. Am Heart J.

[52] Mor-Avi V, Lang RM, Badano LP, Belohlavek M, Cardim NM, Derumeaux G (2011). Current and evolving echocardiographic techniques for the quantitative evaluation of cardiac mechanics: ASE/EAE consensus statement on methodology and indications endorsed by the Japanese Society of Echocardiography. Eur J Echocardiogr.

[53] Fernández A, Politei J (2016) Cardiac manifestation of fabry disease: from hypertrophic cardiomyopathy to early diagnosis and treatment in patients without left ventricular hypertrophy. J Inborn Errors Metab Screen 4:1–9

[54] Pathan F, Zainal Abidin HA, Vo QH, Zhou H, D'Angelo T, Elen E et al (2021) Left atrial strain: a multi-modality, multi-vendor comparison study. Eur Heart J Cardiovasc Imaging 22(1):102–110 10.1093/ehjci/jez303 31848575

[55] Germain DP, Fouilhoux A, Decramer S, Tardieu M, Pillet P (2019). Fila M, et al Consensus recommendations for diagnosis, management and treatment of Fabry disease in paediatric patients. Clin Genet.

