# Supplementary material for: Left atrial strain correlates with severity of cardiac involvement in Anderson-Fabry disease
Source: Eur Radiol. 2022 Nov 2;33(3):2039–51. doi: 10.1007/s00330-022-09183-7 (PMC9935647; doi:10.1007/s00330-022-09183-7)
Supplement: Supplementary file 1 — (DOCX 154 kb) [file 330_2022_9183_MOESM1_ESM.docx]

Supplementary material

| **Table S1.** Stratification of AFD patients at different phases of disease progression | | | | | |
| --- | --- | --- | --- | --- | --- |
|  | **Phase I** | **Phase II** | **Phase III** | **HC** |  |
| n | 19 | 20 | 19 | 50 |  |
| T1 mapping | normal | **↓*** | **↓↓^ꝉ^** | normal |  |
| LVMi | normal | **↑^Ŧ^** | **↑↑^ᶲ^** | normal |  |
| LGE | no | no | present | no |  |
| HC, healthy controls; LVMi, myocardial mass index; LGE, late gadolinium enhancement  ***** cut-offs (calculated as mean_HC_ ± 2 SD): <1101 ms (female), <1080 ms (male)  **^ꝉ^** cut-offs (calculated as mean_HC_ ± 3 SD): <1061 ms (female), <1048 ms (male)  **^Ŧ^** cut-offs (calculated as mean_HC_ ± 2 SD): >65.0 g/m^2^ (female), >80.0 g/m^2^ (male)  **^ᶲ^** cut-offs (calculated as mean_HC_ ± 3 SD): >82 g/m^2^ (female), >89 g/m^2^(male) | | | | | |

| **Table S2.** Calculation of functional parameters of the LA | |
| --- | --- |
| **LA parameters** | **Formula** |
| TEF | $\frac{Vmax-Vmin}{Vmax}\times100$ |
| PEF | $\frac{Vmax-middiastolic Vmin}{Vmax}\times100$ |
| AEF | $\frac{middiastolic Vmax-Vmin}{middiastolic Vmax}\times100$ |
| TEF: Total emptying fraction, PEF: Passive emptying fraction, AEF: Active emptying fraction, Vmax: maximum Volume, Vmin: minimum Volume. | |

| **Table S3.** Frequencies of disease-causing mutations in GLA gene within different phases | | | | |
| --- | --- | --- | --- | --- |
| **Nucleotide** | **Protein change** | **Phase I** | **Phase II** | **Phase III** |
| c.1024C>T | p.R342X | 0 | 2 | 0 |
| c.1163T>C | p.L388P | 1 | 0 | 0 |
| c.1195_1196delTG | p.W399DfsX51 | 0 | 1 | 0 |
| c.119C>T | p.P40L | 1 | 0 | 0 |
| c.119C>T; c.316C>T * | p.P40L; p.L106F | 1 | 0 | 0 |
| c.124A>G | p.M42V | 2 | 1 | 1 |
| c.1250T>C | p.L417P | 0 | 1 | 0 |
| c.128delG | p.G43fs | 0 | 0 | 1 |
| c.334C>T | p.R112C | 0 | 0 | 1 |
| c.352C>T | p.R118C | 1 | 1 | 0 |
| c.392T>C | p.L131P | 0 | 1 | 1 |
| c.410TY>A | p.V137D | 1 | 1 | 0 |
| c.413-414dupG | p.N139kfsX2 | 0 | 0 | 1 |
| c.427G>A | p.A143T | 4 | 0 | 0 |
| c.440G>A | p.G147R | 1 | 1 | 1 |
| c.547+1G>A | p.A143T | 0 | 0 | 1 |
| c.560T>G | p.M187R | 0 | 1 | 0 |
| c.613C>A | p.P205T | 0 | 0 | 1 |
| c.644A>G | p.N215S | 2 | 0 | 0 |
| c.658C>T | R220X | 0 | 0 | 1 |
| c.679C>T | p.R227X | 1 | 1 | 0 |
| c.708G>C | p.W236C | 0 | 2 | 0 |
| c.72G>A | p.W24X | 0 | 2 | 1 |
| c.744delTA | p.F248fs | 2 | 0 | 1 |
| c.803T>C | p.L268S | 0 | 1 | 0 |
| c.806T>C | p.V269A | 0 | 0 | 2 |
| c.899T>C | p.L300P | 0 | 0 | 1 |
| c.901C>T | p.R301X | 1 | 0 | 2 |
| c.902G>A | p.R301Q | 0 | 2 | 0 |
| c.945del21 | p.V316fs | 0 | 0 | 1 |
| c.961C>T | p.Q321X | 0 | 1 | 0 |
| c.973G>A | p.G325S | 1 | 1 | 1 |
| IVS-1G>A | / | 0 | 0 | 1 |
| * female patient | | | | |


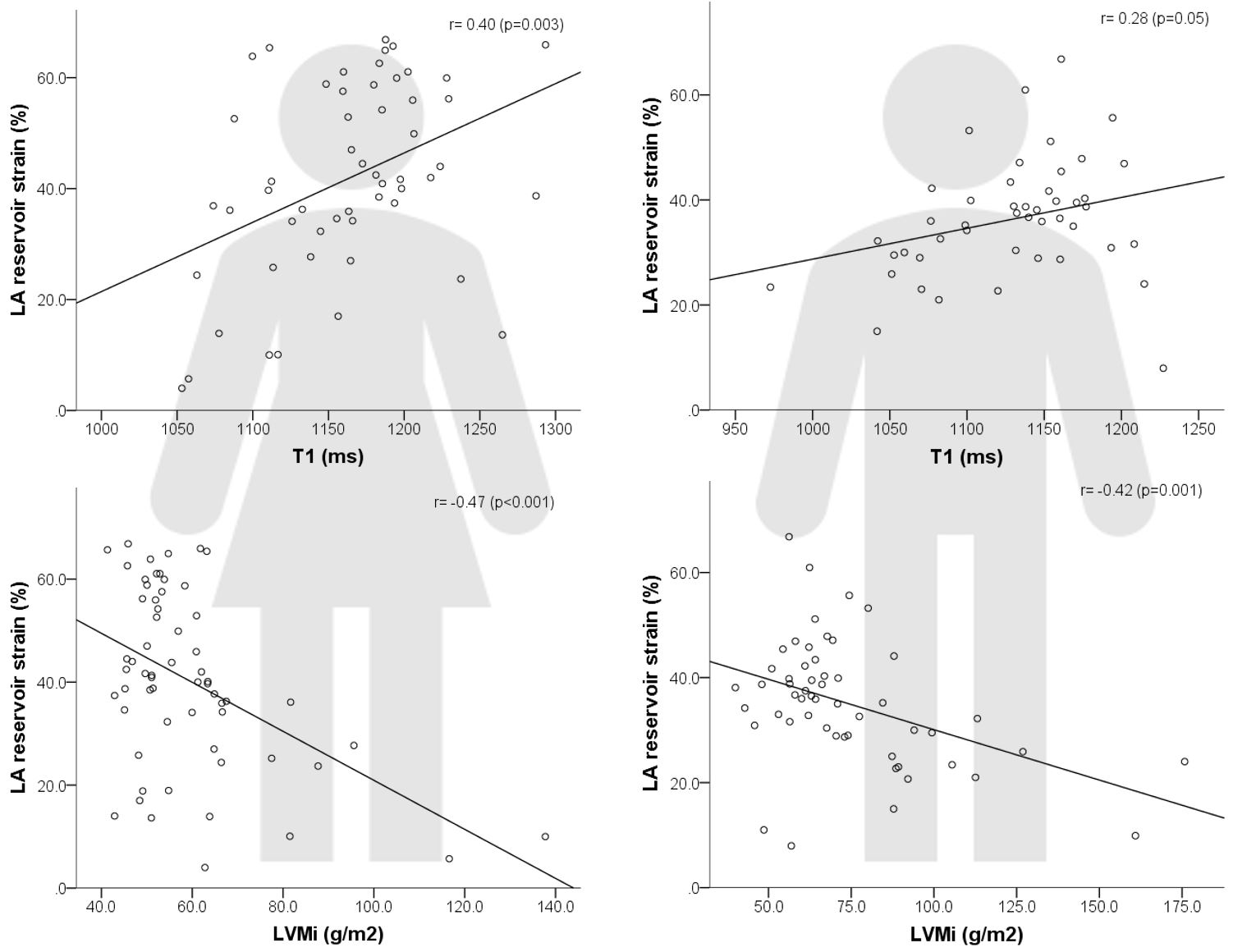


**Figure S4.** Scatter plots illustrating the positive correlation between LA reservoir strain and T1 (top row), and the inverse correlation between LA reservoir strain and LVMi (bottom row), split by sexes (columns).

LA, left atrial; LVMi, left ventricular mass index
